# Pangolin museum genomics reveal the dynamic genetic consequences and extinction risk of the critically endangered Chinese pangolin

**DOI:** 10.1093/molbev/msag099

**Published:** 2026-04-09

**Authors:** Jing Yang Hu, Yu Jiang, Song Li, Ting Ting Ying, Li Yu

**Affiliations:** School of Life Sciences and State Key Laboratory for Conservation and Utilization of Bio-Resources in Yunnan, Yunnan University, Kunming 650091, China; School of Life Sciences and State Key Laboratory for Conservation and Utilization of Bio-Resources in Yunnan, Yunnan University, Kunming 650091, China; Kunming Natural History Museum of Zoology, Kunming Institute of Zoology, Chinese Academy of Sciences, Kunming 650221, China; State Key Laboratory of Genetic Resources and Evolution, Kunming Institute of Zoology, Chinese Academy of Sciences, Kunming 650233, China; School of Life Sciences and State Key Laboratory for Conservation and Utilization of Bio-Resources in Yunnan, Yunnan University, Kunming 650091, China; Southwest United Graduate School, Kunming 650091, China

**Keywords:** temporal dynamics, pangolin, museum genomics, population decline, genetic consequences, extinction risk

## Abstract

Human-driven biodiversity loss, intensified by illegal hunting and trafficking, has caused severe wildlife population declines and extinctions, necessitating studies on long-term genomic erosion to inform conservation strategies. While temporal genomic analyses using ancient and historical/museum DNA reveal generational impacts, sparse sampling often limits insights into prolonged declines, highlighting the need for time-resolved studies to understand sequential population decline and species persistence under sustained pressures. The Chinese pangolin (*Manis pentadactyla*), critically endangered as a result of historical overexploitation, has experienced severe continuous population decline pre-1979, 1980–1999, and post-2000. However, the temporal genetic consequences and associated extinction risks remain poorly understood. We analyzed 228 pangolin genomes (133 newly sequenced), spanning continuous population decline, to assess the dynamics of genomic erosion. Our results demonstrate that persistent population decline drives long-term genetic decline within populations, with the severity of population decline correlating directly with the degree of negative genetic impact (e.g. reduced diversity, increased inbreeding and genetic load) and extinction risk. Counterintuitively, however, between populations, those experiencing the most severe population decline (e.g. southwest China) exhibited less extreme relative genetic consequences compared to less severe population decline (e.g. south China), suggesting a stronger dependence on the history of effective population size before population decline. Critically, the contemporary South China population shows significantly lower genetic diversity, higher inbreeding, elevated genetic load, and consequently higher extinction risk, demanding urgent prioritization for conservation. This study provides novel insights into the complex genomic legacy of continuous population decline, elucidating anthropogenic impacts on genetic erosion and offering a scientific framework for targeted conservation strategies.

## Introduction

Human-driven biodiversity loss, accelerated by illegal hunting and wildlife trafficking, has precipitated catastrophic population declines and contributed substantially to species extinctions globally (Dirzo et al. 2014; Pimm et al. 2014; Ceballos et al. 2015; Finn et al. 2023). Understanding the long-term genomic consequences of such anthropogenic population decline, including erosion of genetic diversity, accumulation of deleterious mutations (genetic load), and intensified inbreeding, is paramount for assessing extinction vulnerability and informing effective conservation strategies (Schwartz et al. 2007). Temporal genomic analyses, leveraging ancient DNA and historical/museum specimens, provide unparalleled insights into how population dynamics shape genomic erosion across generations (Pacioni et al. 2015; Raxworthy and Smith 2021; Irestedt et al. 2022; Jensen et al. 2022; Poo et al. 2022; Benham and Bowie 2023). However, critical limitations persist: many studies rely on sparse historical sampling, often only comparing before and after population decline timepoints or limited post-decline generations, thereby obscuring the temporal trajectory of genetic change over prolonged declines (van der Valk et al. 2019; Dussex et al. 2021; von Seth et al. 2021; Dehasque et al. 2024; Hoelzel et al. 2024). A comprehensive, time-resolved examination of genomic dynamics is thus essential to unravel the compounding effects of sequential population decline and predict the persistence capacity of species under sustained anthropogenic pressures (Dussex et al. 2023).

**Figure 1:**
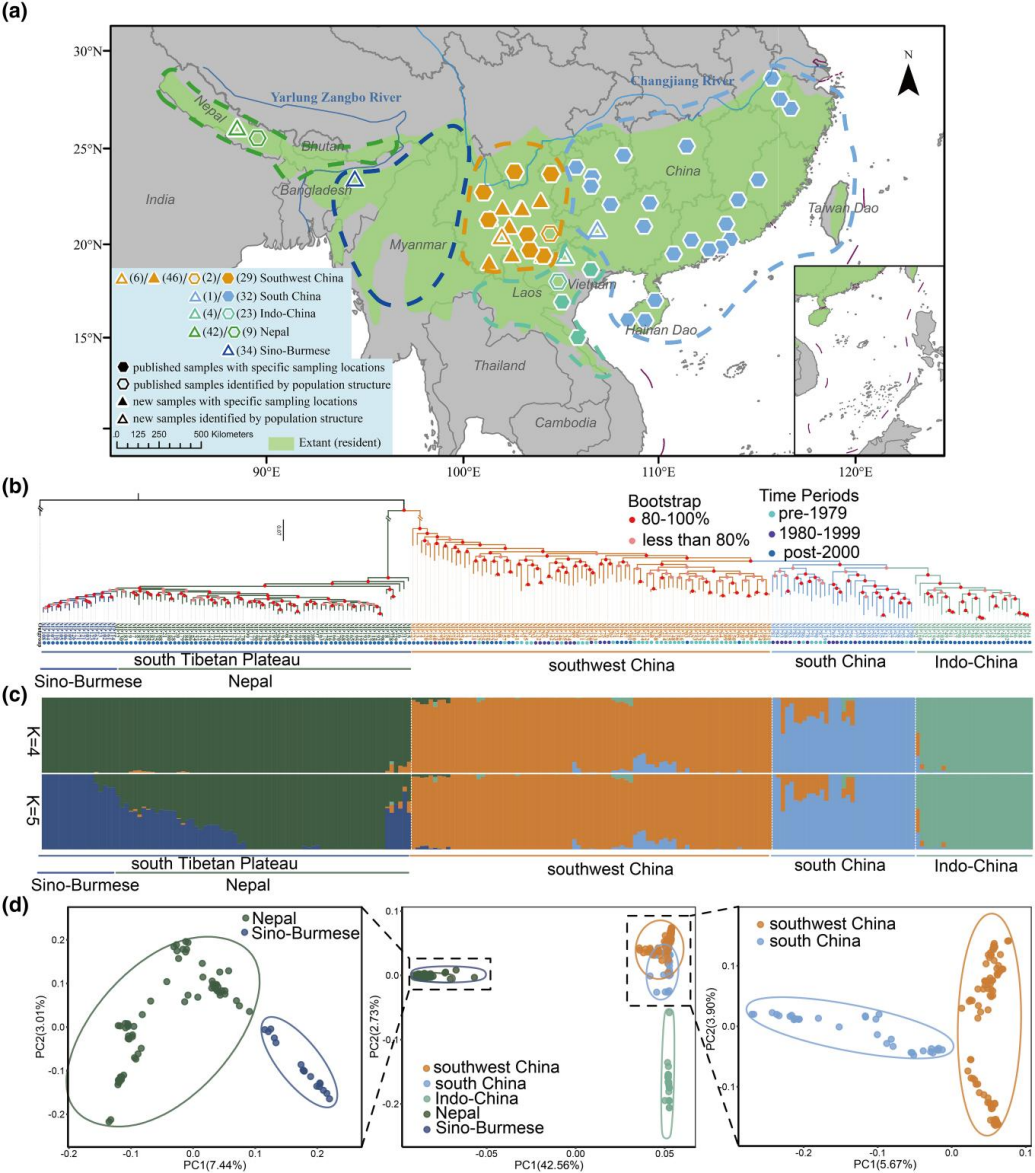
**Sampling information and population structure of museum and contemporary samples of the Chinese pangolin.** (A). The sampling information. A total of 228 Chinese pangolin genomes was obtained, including 133 new obtained samples and 95 published samples. Those with known origins are shown on the map. Triangles represent the new samples and pentagons represent published samples. The samples with specific sampling locations were in solid, while samples with source location identified by population structure were in shape. Different colors represent different genetic populations. The distribution range of extant Chinese pangolin was obtained from the IUCN. (B). Population structure result based on Maximum Likelihood (ML) phylogenetic tree. Bootstrap values and time periods information were listed in the tree using different color-coded circles. Bootstrap values with 80%-100% and <80% are shown red and orange color in the branch. Time periods of pre-1979, 1980–1999 and post-2000 samples are shown green, purple and blue sample names, respectively. (C). Admixture results with K values ranging from 4–5, and supported five ancestral populations (K = 5). (D). Principal component analysis (PCA) of the Chinese pangolin. The dark green, dark blue, orange, light blue, and light green represent the Sino-Burmese, Nepal, southwest China, south China, and Indo-China populations of the Chinese pangolin, respectively.

The Chinese pangolin (*Manis pentadactyla*), a critically endangered mammal, historically and presently endemic to southern China, northern Southeast Asia, and northern South Asia (Fig. 1a), epitomizes this crisis. The Chinese pangolin exhibits a slow life-history strategy, characterized by a low reproductive output (typically one offspring per litter, with females giving birth once per year or less), and limited dispersal capacity (Challender et al. 2020). These traits, combined with their specialized myrmecophagy (Cheng et al. 2022) and vulnerability to habitat fragmentation (Peng 2020) may lead to slower population recovery and higher extinction risk. The population size of Chinese pangolin was approximately 800,000 in the 1960s (Wu et al. 2004; Zhang et al. 2010; Peng 2020), after which it has been continuously declining due to the relentless poaching and trafficking (Heinrich et al. 2016; Challender et al. 2020; Zhang et al. 2022). The population was estimated at ∼64,000 in the late 1990s (Wang and Xie 2004; Wu et al. 2004; Zhang et al. 2010) and ∼16,000 in 2020 (Peng 2020). These estimates indicate substantial declines across two-time periods: a sharp reduction ∼90% between the 1980s and 1990s (representing a loss of approximately 736,000 individuals) and a further decline of ∼75% from 2000 to 2020 (representing a loss of approximately 48,000 individuals). Overall, the Chinese pangolin population has experienced sustained declines since the 1960s, occurring in two periods that differed in severity and pace. We define the period from pre-1979 to 1980–1999 as the period I of population decline, and the period from 1980–1999 to post-2000 and beyond as the period II. Yet, despite this severe, multi-decadal decline spanning more than 60 years/generations (considering a generation time of one year; Zhang et al. 2016; Challender et al. 2020; Heighton et al. 2023), genetic assessments remain strikingly limited. Existing studies mainly describe contemporary genetic patterns or lack the temporal coverage required to infer genomic change through time (Hu et al. 2020; Wang et al. 2022; Wei et al. 2024; Lan et al. 2025; Zhai et al. 2025). However, the availability of well-preserved historical specimens spanning periods of severe decline provides an unusual opportunity to examine genomic trajectories in Chinese pangolins. This unique scenario enables an unprecedented investigation into: (1) the dynamics of genetic diversity erosion across discrete decline phases; (2) the tempo and mode of genetic load accumulation under low population size; and (3) the compounding genomic impacts of continuous population decline.

We therefore integrated temporal genomics from museum and modern Chinese pangolin samples, spanning the periods of continuous population decline, to explore anthropogenic impacts on genetic erosion. Based on the analyses of 228 pangolin genomes (133 newly sequenced), this study provides novel insights into the genomic legacy of continuous population decline, elucidating anthropogenic impacts on genetic erosion and offering a scientific framework for targeted conservation strategies of this critically endangered species.

## Results

### Sample information and genomic dataset

We analyzed 228 Chinese pangolin genomes, including 133 newly generated in this study and 95 from previously published datasets. These samples comprise 23 individuals collected pre-1979, 51 individuals collected in 1980–1999, and 154 individuals collected post-2000 (Table S1). Among them, 133 genomes were newly obtained, achieving an average clean data depth of 19.04× (ranging from 10.93× to 48.21×), an average mapping depth of 11.43× (ranging from 6.04× to 32.72×), and an average coverage of 98.17% (ranging from 91.01% to 99.52%) (Table S1). The previously published 95 genomes (including 9 individuals from south Tibetan Plateau, 23 individuals from Indo-China, 31 individuals from southwest China, and 32 individuals from south China) with an average clean data depth of 33.14× (ranging from 11.92× to 63.15×), an average mapping depth of 17.58× (ranging from 6.42× to 34.70×), and an average coverage of 98.57% (ranging from 89.47% to 99.84%) (Table S1; Hu et al. 2020; Wang et al. 2022; Wei et al. 2024; Zhai et al. 2025). DNA damage analysis showed that samples from pre-1979, 1980-1999 and post-2000 had ranges of C (Cytosine) to T (Thymine) substitution frequencies, at the first bp on the 5′ end, of 0.35% to 2.02%, 0.17% to 2.81%, and 0.11% to 0.91%, respectively (Figure S1a). The ranges of G (Guanine) to A (Adenine) substitution frequencies at the first bp on the 3′ end were 0.01% to 1.48%, 0.03% to 2.02%, and 0.28% to 1.11%, respectively (Figure S1b). There were no significant differences in the substitution frequencies from C to T and G to A among the samples from across the three periods, indicating that the museum samples could be utilized for subsequent genetic analyses (Figure S1c and S1d). Ultimately, we obtained a dataset of 105,229,880 high-quality single-nucleotide polymorphisms (SNPs).

### Population structures

The previously published genomes were used to build a reference panel, and all the individuals were assigned to four populations (south Tibetan Plateau, Indo-China, southwest China, and south China), as defined by Wei et al. (2024) and Zhai et al. (2025) (Fig. 1), based on phylogenetic analysis, admixture analysis, and principal component analysis (PCA). Compared to previous studies (e.g. Wei et al. 2024; Zhai et al. 2025), by integrating our newly generated samples from the south Tibetan Plateau (STP), our population structure analysis revealed an important refinement. Specifically, STP showed further divergence into two distinct subpopulations: a Nepal subpopulation and a Sino-Burmese subpopulation (Fig. 1b-1d). Given that the samples from the south Tibetan Plateau and Indo-China populations were obtained from recent confiscations and no historical specimens are available, these two populations were not included in the analyses of historical demographic trends or genetic consequences over time.

### Temporal dynamics of effective population size

**Figure 2:**
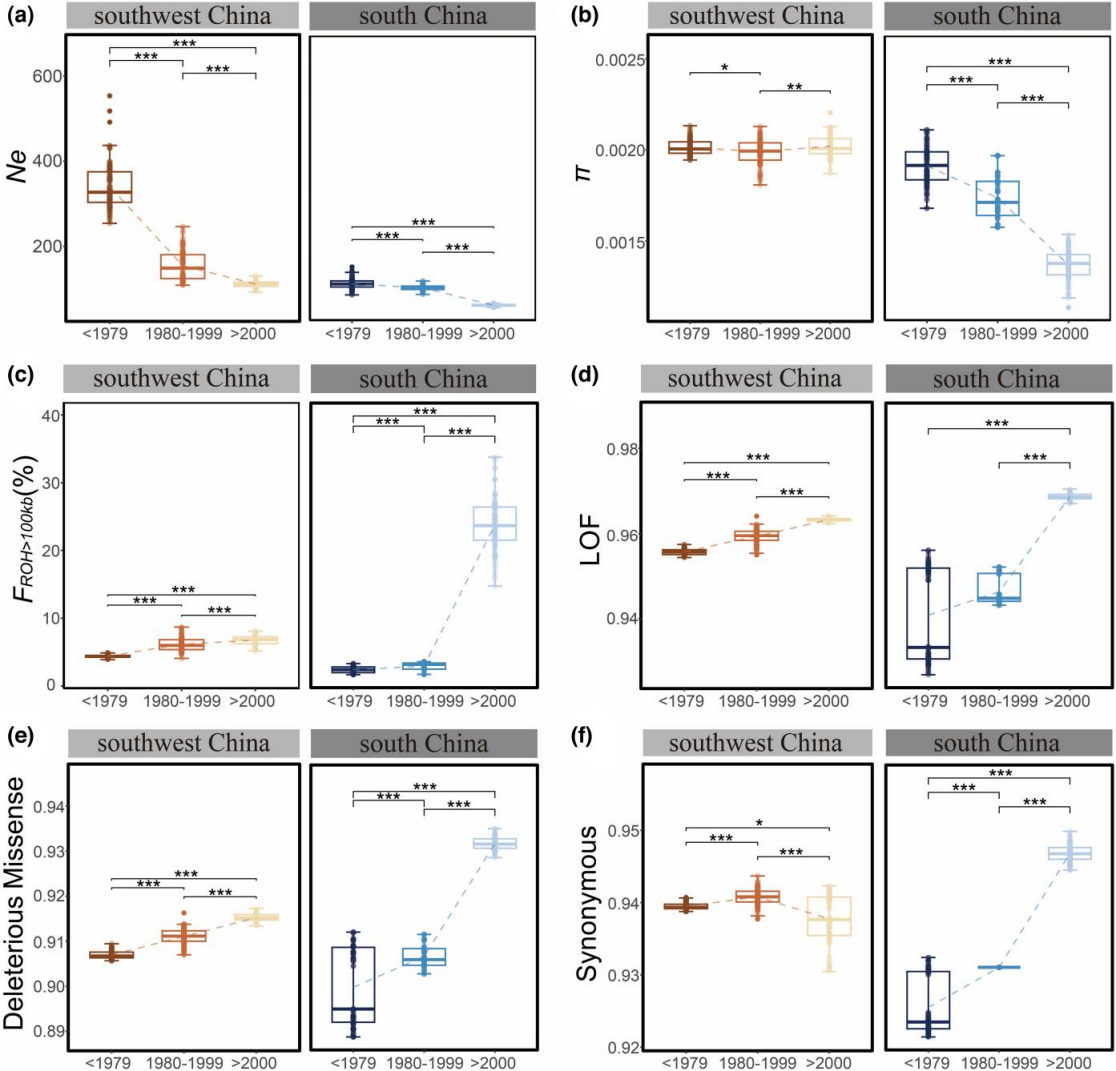
**Dynamic genetic consequences of southwest China and south China populations of pangolin**. (a) The effective population size (*N*e) conducted 100 estimations, (b) nucleotide diversity (*π*), (c) inbreeding coefficient (*F*_ROH > 100kb_), (d) genetic load of loss of function (LOF) mutations, (e) genetic load of deleterious missense mutations, and (f) genetic load of synonymous mutations. The dashed lines illustrate the trend in the median. Each genetic consequence was resampled 100 times. All the results were subjected to significance testing using the Wilcoxon rank-sum test method. ***, **, and * represent *P* values less than 0.001, less than 0.01, and less than 0.05, respectively.

Notably, both the southwest China and south China populations comprised samples spanning all three periods (pre-1979, 1980–1999 and post-2000). Sample numbers of these three-time period for the southwest China and south China populations were 10, 7, 16, and 13, 44, 26, respectively. We conducted 100 estimations of the effective population size (*N*e) for each of the two populations over the three time periods, and found that the *N*e decline of the southwest China population between the pre-1979 and 1980–1999 (period I; 54.63% decline) was more pronounced than between 1980–1999 and post-2000 (period II; 27.93% decline) (Fig. 2a). In contrast, the South China population experienced a more significant decline in period II, with a decrease of 40.81% compared to 8.59% in period I (Fig. 2a). Notably, the *N*e of the southwest China and south China populations decreased in total by 67.30% and 45.89%, respectively, after a sequential population decline (Fig. 2a).

### Temporal dynamics of genetic consequences

The temporal dynamics of the genetic consequences were compared across different time periods (Fig. 2b and Table S2-S3). We found no significant change in genetic diversity in the southwest China population (Wilcoxon rank-sum test, not significant), whereas the south China population showed a statistically significant decrease of 28.56% (Wilcoxon rank-sum test, *P* value < 0.001). The southwest China population exhibited a slight decline in genetic diversity of 1.12% in period I (Wilcoxon rank-sum test, *P* value < 0.05), followed by a slight increase of 1.46% in period II (Wilcoxon rank-sum test, *P* value < 0.01). Conversely, the South China population experienced a more substantial loss of genetic diversity, with a 9.23% loss in period I (Wilcoxon rank-sum test, *P* value < 0.001) and a significantly greater decline of 21.30% in period II (Wilcoxon rank-sum test, *P* value < 0.001). Furthermore, we examined the genetic consequences in relation to the precise sampling time of each individual (Figure S2). This analysis revealed that the temporal patterns (including decreasing genetic diversity, increasing inbreeding levels, and increasing genetic load) in both populations were correlated with sample collection dates. These patterns are consistent with the results obtained using the discrete time-period division.

Additionally, we compared the admixture across different datasets based on sampling time (Figure S3a). The results suggested historical gene flow between the southwest China population and the South China population. To explicitly determine whether the observed genetic differences were primarily driven by gene flow between populations versus genetic changes within populations over time, we used the proportion of mixed individuals within each period to characterize relative gene flow intensity over time (Figure S3). We observed an increasing (though statistically non-significant) trend in estimated gene flow from the pre-1979 to the post-2000 period in both populations, which was more pronounced in the South China population. Crucially, when we examined genetic diversity trends, in the south China population, genetic diversity significantly decreased from the pre-1979 to post-2000 periods for both mixed and non-mixed individuals; in the southwest China population, genetic diversity showed no significant change over time for either group. Therefore, the parallel decline in diversity among both mixed and non-mixed individuals in the South China population suggests that the dominant signal in our data reflects temporal genetic change within the population, rather than a pattern attributable solely to the inclusion of mixed individuals.

Decreased genetic diversity was frequently associated with increased inbreeding levels (Sánchez-Barreiro et al. 2021; de Ferran et al. 2022). The genomic inbreeding coefficient (*F*_ROH > 100kB_) indicated that the inbreeding levels in both populations showed similar patterns to the genetic diversity (Fig. 2c). The same results are obtained when the length of ROH is divided into 100 kb to 1 Mb and length longer than 1 Mb (Figure S4). The increase in inbreeding level of the southwest China population in period I (31.92%) was larger than period II (9.73%) (Wilcoxon rank-sum test, *P* value < 0.001). In contrast, the south China population experienced a 23.30% increase in inbreeding level in period I, significantly lower than the 741.45% (i.e. approximately seven times) increase in period II (Wilcoxon rank-sum test, *P* value < 0.001). Over all three periods, we found a significant increase in inbreeding level in the southwest China population (53.91%) (Wilcoxon rank-sum test, *P* value < 0.001), while the south China population exhibited a significant increase (937.53%, i.e. approximately nine times) (Wilcoxon rank-sum test, *P* value < 0.001).

Higher inbreeding levels can result in a higher genetic load, which may reduce the adaptive potential of the current environment and diminish individual fitness (Charlesworth and Willis 2009; Hu et al. 2020). The genetic load of loss of function (LOF) mutations, deleterious missenses, and synonymous mutations, between period I than period II in the southwest China population increased by 0.39% and 0.41%, 0.45% and 0.46%, and 0.14% and 0.33%, respectively, and in the south China population by 0.59% and 2.37%, 0.77% and 2.78%, and 0.06% and 1.70%, respectively (Fig. 2d–2e). From the pre-1979 to the post-2000 period, genetic load increased in the South China populations, with the three types rising by 2.97%, 3.57%, and 2.32%, respectively. Concurrently, the southwest China population exhibited an increase of 0.80% in LOF mutations and 0.91% in deleterious missense mutations, while synonymous mutations showed a decrease of 0.19% (Fig. 2d–2e). The pattern of increased genetic load was consistent with decreased genetic diversity and increased inbreeding level, which supported the south China population having a higher rate of genetic load in period II than period I.

Although the decrease in genetic diversity was not significant in the southwest China population from pre-1979 to post-2000, the inbreeding level, genetic loads of LOF mutations, and deleterious missense mutations significantly increased (Fig. 2b–2e, Wilcoxon rank-sum test, *P* value < 0.001). Additionally, the temporal dynamics of genetic consequences in the south China population were shown to be more significant in period II than period I, as well as between pre-1979 and post-2000 (Fig. 2b–2f, Wilcoxon rank-sum test, *P* value < 0.001).

### Extinction risk and fitness recovery

**Figure 3:**
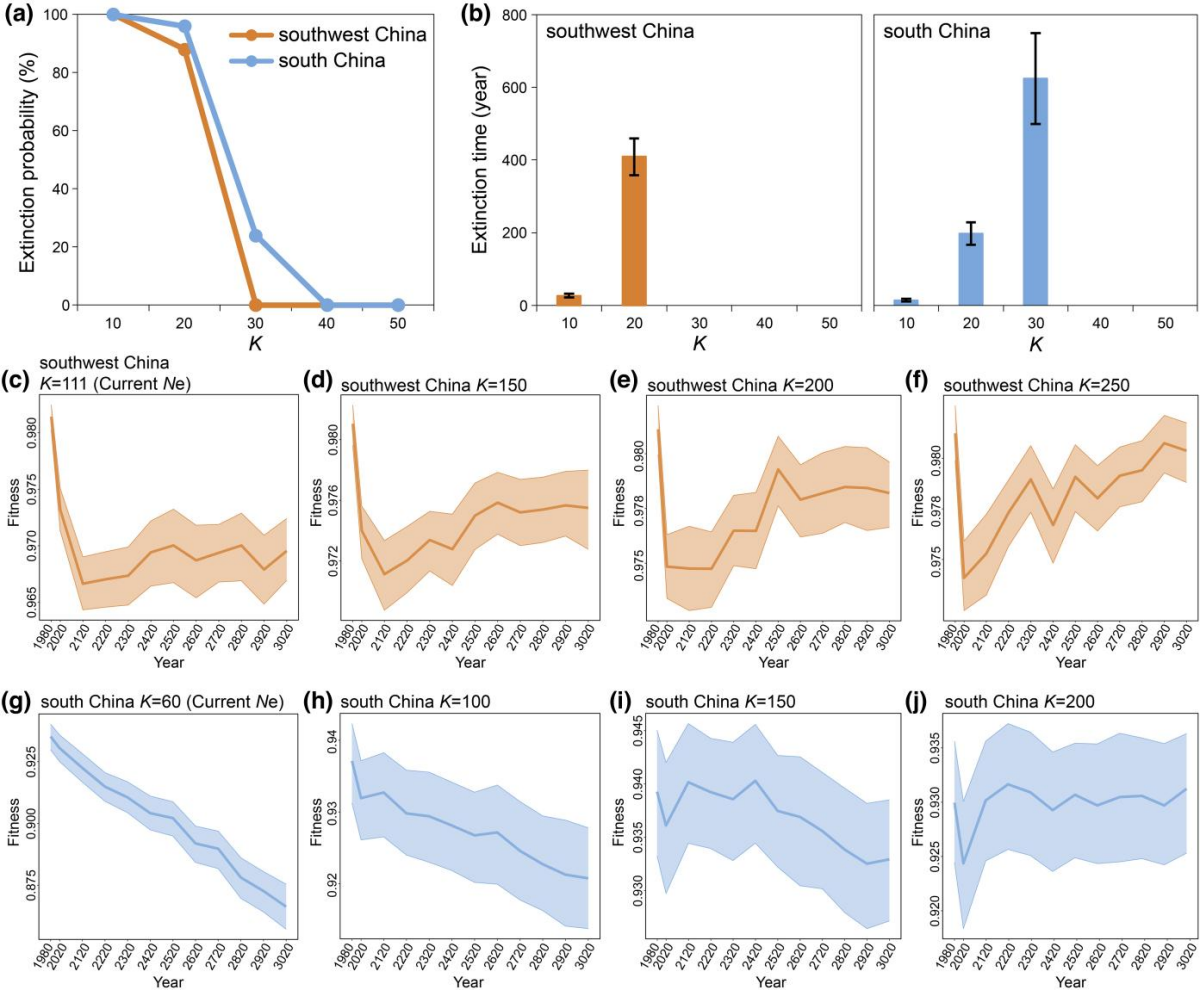
**Non-Wright–Fisher simulations of southwest China and south China pangolin populations**. (a) Extinction probabilities. The *x*-axis represents the different carrying capacities (k) for the next 1000 years. The *y*-axis represents the probabilities of extinction in 100 simulations. (b) Extinction times. The upper and lower limits of the column charts are standard error lines. (C)–(F) Fitness of the southwest China population at *K* = current *N*e (C), *K* = 150 (D), *K* = 200 (E), and *K* = 250 (F). The fitness will recover by 0.15%, 0.34%, and 0.64% if the *K* increases by 150, 200, and 250, respectively. (G)–(J) Fitness of the south China population at *K* = current *N*e (G), *K* = 100 (H), *K* = 150 (I), and *K* = 200 (J). The degree of decline in fitness has diminished from 1.79% at *K* = 100 to 0.34% at *K* = 150, and reaches equilibrium (0% change) at *K* = 200.

To simulate the extinction risk facing the southwest China and south China populations over the next 1000 years with a pessimistic scenario by setting a carrying capacity (*K*) value from 10 to 50. We found that the extinction probabilities of both populations would gradually increase, followed by a decrease in *K* value (Fig. 3a). Our results indicated critical viability thresholds at present: the Southwest China population was at high risk of extinction below *K* < 30, while the South China population faced extinction below *K* < 40 (Fig. 3a). For the southwest China population, extinction probability reached 88% within 409 years at *K* < 20 and 100% within just 27 years at *K* < 10. Similarly, the south China population showed a 24% extinction risk within 625 years at *K* < 30, escalating to 96% within 200 years at *K* < 20 and 100% within 17 years at *K* < 10, with more severe declines leading to drastically earlier extinction dates (Fig. 3a, b). Moreover, we observed that the south China population exhibits a higher extinction probability and an earlier extinction time compared to the southwest China population with a *K* less than 30, suggesting that the South China population faces a greater extinction risk under equivalent survival pressures (Fig. 3a and 3b).

We also conducted more optimistic simulations for both populations (Figs 3c–3j). The fitness of the population was negatively correlated with the genetic load, which reflected the survival conditions of both populations over the next 1000 years. Under *K* equal to current *N*e conditions, the southwest China population exhibits stable fitness after a sharp decline until around 2120 (Fig. 3c). However, the fitness will recover by 0.15%, 0.34%, and 0.64% if the *K* increases by 150, 200, and 250, respectively (Figs 3d–3f). Conversely, the fitness of the South China population is projected to continue declining if *K* is maintained at the current *K* (Fig. 3g). However, the degree of decline in fitness diminished with increasing *K*, eventually stabilizing. Specifically, the degree of decline in fitness has diminished from 1.79% at *K* = 100 to 0.34% at *K* = 150, and reaches equilibrium (0% change) at *K* = 200 (Figs 3h–3i).

## Discussion

Based on the largest-scale population genetic analysis of Chinese pangolins to date (n = 228), we identified five genetic clusters, revising the previously recognized structure of four populations (Fig. 1b–1d; Wei et al. 2024; Zhai et al. 2025). Prior studies (Hu et al. 2020; Wei et al. 2024; Zhai et al. 2025) broadly assigned confiscated samples from the China–Myanmar border region to the South Tibetan Plateau (STP) population. Our analysis now refines this assignment, placing all such confiscated samples specifically within the Nepal subpopulation. This may indicate that seized individuals in this border area likely originated specifically from the Nepal portion of the STP range, offering more precise forensic tracing for wildlife law enforcement. Furthermore, the recognition of two distinct subpopulations within the STP underscores the need for separate conservation strategies for the Nepal and Sino-Burmese groups, as they may face differing threats or demographic trajectories. Thus, our refined substructure not only clarifies the geographic origins of confiscated samples but also provides actionable information for conservation management.

Notably, this study has utilized a large population genomics dataset for southwest China and south China populations of Chinese pangolins (from museum and contemporary samples) to assess the genetic legacy of decline over the past 60-plus years/generations, and evaluate future extinction risk. Our analysis reveals novel insights into the genetic complexities underlying continued population decline. We confirmed genetically that the Chinese pangolin distributed in China has been declining since the 1960s, with two major population declines in 1980–1999 and post-2000 (Wang and Xie 2004; Wu et al. 2004; Zhang et al. 2010; Peng 2020). The first significant decline (1980–1999) was likely driven by large-scale commercial hunting, fueled by strong demand for consumption and medicinal use, in the absence of legal protections and widespread conservation awareness (Wu et al. 2004; Zhang et al. 2010). Although pangolins were listed in Appendix II of CITES in 1995, classified as Endangered by the IUCN in 1997, and designated as a Class II protected species in China, these conservation measures have been undermined by soaring black-market prices and the enormous profits from illegal trade (Wu et al. 2004; Zhang et al. 2010; Peng 2020). Consequently, poaching and smuggling have persisted despite repeated bans, leading to another significant population decline, albeit at a slower rate than the previous one. However, the extent of population decline differs between the southwest China and south China populations across these two time periods (Fig. 2a). The southwest China population experienced the most significant decline during 1980–1999, while the south China population showed a more pronounced reduction in the post-2000 period. Meanwhile, genetic consequence dynamics intensified with the severity of population decline, the southwest China population exhibiting greater losses of genetic diversity, higher increases in inbreeding level, and more substantial accumulation of genetic load during the 1980–1999 period, after greater population decline severity compared with the post-2000 period. The south China population also displayed more negative genetic consequences after greater population decline severity during the post-2000 period compared with 1980–1999 (Figs 2b–2f). Our results suggested that population reduction was more severe in southwest China during 1980–1999, probably as a result of human activities such as hunting and habitat fragmentation, and poor management of the nature reserves distributed in southwest China at that time (Liu et al. 2001; Xu and Melick 2007). In addition, the south China population showed a more severe population decline in the post-2000 period, further supporting a markedly higher human population and intensified land use as the main causes of pangolin population shrinkage and more serious genetic consequences in south China (Wei et al. 2024).

In general, the increased gene flow is typically expected to enhance genetic admixture, reduce differentiation, and potentially increase *Ne*. In our study, although we detected signals of increased gene flow, these signals were not statistically significant (Figure S3). This indicates that while some genetic exchange may be occurring, it is likely limited to a small subset of individuals and is too weak to drive population-level changes in genetic diversity or reduce inbreeding. In addition, even if some gene flow has occurred recently, a longer period may be required for its effects to meaningfully reduce the existing inbreeding load or increase *Ne*. Moreover, the key driver of the high inbreeding here is more likely to be the rapid and severe population decline. The population size has become so small that genetic drift and forced mating among relatives have overwhelmed any potential benefits from the limited gene flow. In other words, the signal of inbreeding is a consequence of population contraction, which is a much stronger force in this context. In summary, our results suggest that the magnitude of gene flow was insufficient to counteract the strong and rapid genetic consequences of population decline.

The temporal dynamics of genetic consequences revealed the Chinese pangolin has experienced severe genetic erosion in each population, especially after the more severe population decline (Fig. 2). Globally, many species have undergone severe population declines during the past century, resulting in significant genomic consequences. For instance, the eastern gorilla (*Gorilla beringei*) has experienced a 20% loss of genetic diversity (van der Valk et al. 2019), the northern and southern white rhinoceros (*Ceratotherium simum*) have experienced significant genetic diversity decreases of 10% and 36% (Sánchez-Barreiro et al. 2021), respectively, and the koala (*Phascolarctos cinereus*) has experienced a 30% reduction (De Cahsan et al. 2025). Similar to pangolins, the crested-ibis (*Nipponia nippon*) has suffered even more pronounced consequences because the genome has changed more rapidly as a result of shorter generation times, with a significant decrease in genetic diversity of about 46.15% (Feng et al. 2019). More severe population decline typically exhibits lower genetic diversity and higher inbreeding levels, as reported for the brown-eared pheasant (*Crossoptilon mantchuricum*) and blue-eared pheasant (*C. auritum*) (Wang et al. 2021). Similarly, the northern elephant seal (*Mirounga angustirostris*) has experienced a stronger population decline than the southern elephant seal (*M. leonina*), showing higher levels of reduced genetic variation and increased inbreeding (Hoffman et al. 2024).

Remarkably, although the southwest China population has experienced a more severe population decline compared to the south China population (67.30% *vs* 45.89%), it has been less affected by negative genomic consequences (genetic diversity 0.05% *vs* 28.56%; inbreeding level 59.49% *vs* 1078.97%; LOF mutations 0.79% *vs* 2.97%; deleterious missenses 0.91% *vs* 3.57%; synonymous mutations 0.19% *vs* 2.32%) (Fig. 2). However, the southwest China population has a larger historical *N*e than the south China population (Fig. 2a). Therefore, our results indicate that, compared with the strength of population decline, historical *N*e plays a decisive role in the magnitude of genetic diversity decline and increase in inbreeding level among different Chinese pangolin populations. In addition, compared with the southwest China population, the south China population experienced faster increasing genetic load during sequential population decline (Fig. 2d–2f). This aligned with the theory that populations with smaller historical *N*e exhibit higher genetic loads (van Oosterhout 2020; Kardos et al. 2021; Dussex et al. 2023). Our findings demonstrated that the severity of population decline did not necessarily correlate positively with extinction risk. Populations with smaller historical *N*e faced higher extinction risks, yet paradoxically tended to be neglected in current conservation frameworks. Therefore, we recommend that species threat assessments should incorporate multiple parameters, including historical *N*e, the strength of population decline, and genetic consequences, for more robust conservation prioritization.

Additionally, the simulation results indicated that for both the southwest China and south China populations of the Chinese pangolin, extinction probability gradually increased as the carrying capacity decreased under scenarios of continued population decline. Conversely, fitness could recover with an increase in carrying capacity if proactive and effective conservation measures were implemented to facilitate population size growth over the next 1000 years (Fig. 3). These findings underscore that maintaining or increasing population size is a key determinant of long-term viability for small, endangered populations (Benham and Bowie 2023; Dehasque et al. 2024). Population decline is associated with reduced genetic diversity and increased genetic load, which compromise adaptive potential and population resilience, thereby elevating extinction risk (van der Valk et al. 2019; Irestedt et al. 2022). In contrast, population growth can alleviate genetic constraints by expanding effective population size, slowing genetic drift, enabling the purging of deleterious mutations, and promoting fitness recovery (Hoelzel et al. 2024; Hoffman et al. 2024). Although current population trends are concerning, this study also demonstrates that targeted, science-based conservation interventions can effectively alter these trajectories. Therefore, it is crucial to enhance and expand the management of existing protected areas, strengthen collaboration with law enforcement, and intensified efforts to combat poaching and illegal trade. Furthermore, habitat restoration initiatives should be implemented in key regions to scientifically mitigate habitat fragmentation. These measures should be supported by strengthened fundamental research into pangolin ecology, disease, and reproductive biology, which will help advance captive breeding programs and promote population recovery.

Notably, the south China population has a smaller effective population, worse genetic consequences, and a higher extinction risk compared with the southwest China population (Figs 2 and 3). Therefore, it is vital that priority is given to protecting the South China population. In 2020, the Chinese government established the Chinese Pangolin Conservation and Research Center in Guangdong, southern China, and elevated the species to a first-class national protected animal. These efforts were expected to strengthen the protection measures for Chinese pangolins and promote the recovery and growth of the south China population (Zhang et al. 2021). However, the imbalance in the allocation of resources for protection between South China (high investment) and the southwestern (lower investment) regions might create inaccurate distribution patterns through differential survey intensity rather than actual ecological preferences. Multiple lines of evidence (2010–2023), from literature, interviews, and field observations, have established that the individuals from the southwest China population have rarely been recorded compared to the south China population, which has larger populations of extant Chinese pangolins (Peng 2020; Zhang et al. 2021; Liu et al. 2024). However, our newly acquired samples from southwest China population, collected between 2010 and 2024 (Table S1), were primarily sourced from illegal trade activities. This suggests that the southwest China population may be linked to transboundary regions such as Myanmar. The scarcity of survey data along the China-Myanmar border likely results in an incomplete assessment of this population's status. Therefore, future conservation strategies should not only sustain strong protection of the well-documented south China population but also expand efforts to include comprehensive surveys across the species' entire range, with particular emphasis on the southwest China population. The recommendations also included enhancing protection in underrepresented western regions and strengthening international collaboration for transboundary conservation.

Long-term genetic monitoring of endangered species can effectively assess population viability and inform critical conservation strategies, as demonstrated in studies of island foxes and gorillas (van der Valk et al. 2019; Adams and Edmands 2023). Conservation planning must account for substantial differences in population history, genetic consequences, survival potential, and extinct risks among different Chinese pangolin populations. Therefore, we recommend that long-term genetic monitoring be conducted for different populations of Chinese pangolins in the future. This will allow timely assessment of the recovery status of Chinese pangolins across different regions and facilitate targeted conservation efforts.

In conclusion, by integrating temporal genomics from museum and modern Chinese pangolin samples, we have revealed that the anthropogenic impacts on genetic erosion are direct and consistent, but the complexity lies in the pathways where several factors (historical *Ne*, number of population decline, the duration and severity of population decline, and the ability of individuals to respond or adapt) interact and compound to produce the outcomes. While inter-population genetic consequences correlate directly with the severity of population decline, intra-population differences were primarily driven by the historical effective population size, not the intensity of population decline. Critically, the south China population shown severely reduced genetic diversity, higher inbreeding, and greater extinction risk than the southwest China population, calling for prioritized conservation. More generally, this work establishes a framework for science-based preservation of critically endangered species.

## Materials and methods

### Sample collection

One-hundred and thirty-three new Chinese pangolin samples, including scale, skin and muscle, were collected from the Kunming Natural History Museum of Zoology, Kunming Institute of Zoology Wild Germplasm Bank, and Yunnan Forest Public Security Bureau. All necessary research permits and ethical approvals were obtained (YNU20240775). All skin samples were obtained from distinct preserved specimens housed at the Kunming Natural History Museum of Zoology. Each muscle sample was sourced from the Kunming Institute of Zoology Wild Germplasm Bank, with recorded information confirming that they originated from different individuals. For scale samples, we deliberately selected scales from separate confiscation batches that represented different haplotypes, all provided by the Yunnan Forest Public Security Bureau, thereby minimizing the possibility of sampling the same individual. Published resequencing data from 95 Chinese pangolins (Hu et al. 2020; Wang et al. 2022; Wei et al. 2024; Zhai et al. 2025) were also analyzed, using the white-bellied pangolin as the outgroup (Gu et al. 2023). Detailed information about the samples can be found in Table S1.

### DNA extraction and whole genome resequencing

To eliminate preservatives from the museum samples, they were rinsed three times with 70% alcohol. A HiPure Tissue DNA micro-kit (Mega, China) was used to extract DNA. The extracted DNA was detected by gel electrophoresis using a 1% agarose gel and Lambda DNA/HindIII Marker, and quantified with a Nanodrop 2000 spectrophotometer. Samples with an extraction volume exceeding 100 ng/µL were sequenced based on a constructed 500-bp Illumina short fragment library, and 150-bp paired-end resequencing performed on an Illumina NovaSeq 6000 platform. We removed splice sequences from the original resequencing data, and filtered out reads with more than 20% low-quality bases and more than 10% N-bases. The newly obtained resequencing data have been uploaded to CNGBdb (accession number CNP0007284).

### Read alignment and variant calling

We used the chromosome-level Chinese pangolin genome as the reference genome (Lan et al. 2025). The software BaseNumber DNA v.1.0.3 (Zhang et al. 2021), which uses a high performance GPU-accelerated variant-calling method, was utilized for read alignment and to obtain the whole genome single nucleotide polymorphisms (SNPs), using a default setting. We employed the “slaidx” command to establish the index of the reference genome, and used the “sla” command to map all reads to the reference, and obtained a BAM file for each sample. We utilized mapDamage v.2.0 (Jónsson et al. 2013) with a default setting to estimate the DNA damage of each final BAM file, including museum samples and other samples, which covered different collection times. The “slc” command was then used to detect mutations based on each BAM file, and all variant information saved into a GVCF file. The GVCF file for all the samples was used with the “slmgvcf” command to merge and convert the final GVCF file to a VCF file using the “slgtvcf” command. We used GATK v.4.1.2.0 (McKenna et al. 2010) to extract and filter the SNPs for all individuals. The filtering criteria were as follows: QUAL < 30.0, QD < 2.0, MQ < 40.0, FS > 60.0, SOR > 3.0, MQRankSum<–12.5, ReadPosRankSum<–8.0, SB≥–1.0, DP < 3, MQ0 ≥ 4 and MQ0/DP ≥ 0.1. We retained SNPs on 19 autosomes based on the chromosome-level reference genome using VCFtools v.0.1.13 (Danecek et al. 2011). We excluded SNPs with missing site coverage greater than 20% for all samples, and ensured a diploid count of two alleles within the dataset. We removed SNPs with sequencing depths less than 2.5% depth distribution or greater than 97.5% depth distribution, to reduce potential false positive sites in the sequencing. Kinship analysis (Manichaikul et al. 2010) was performed on the genomic data to further confirm that all newly obtained skin, muscle, and scale samples were derived from distinct individuals.

### Population structure analysis

We employed phylogenetic, admixture, and principal component analyses (PCA) to characterize the population structure of Chinese pangolins and assign museum specimens and confiscated samples to their respective populations. To minimize the influence of linkage disequilibrium (LD) on population structure, we utilized VCFtools to disrupt the LD by selecting one site every 10 kb. This process ultimately yielded 235,144 SNPs for the population structure analysis. We constructed a maximum likelihood (ML) phylogenetic tree using RAxML v.8.2.12 (Stamatakis 2014), employing the GTRGAMMA model with 1000 bootstrap replicates. We used Dystruct (Joseph and Pe’er 2019), a method applicable to infer population structure from time-series samples, to reanalysis the population admixture. We converted the sampling time of all samples into sampling time period, assuming a sampling time period of 20 years. Based on these input files, we performed admixture analysis using Dystruct v.1.1.0. We gradually increased the assumed number of genetic clusters (K) from one to ten. When using Dystruct, we applied the parameter –hold-out-fraction 0.1 for comparison between different K values, and the K value with the highest hold-out log likelihood was determined as the optimal one. For the PCA, we utilized the smartPCA program in Eigensoft v.4.2 (Patterson et al. 2006), selecting the first principal component (PC1) and the second principal component (PC2) that contributed most significantly to evaluating the genetic relationships among individuals.

### Population dynamics, genetic diversity, inbreeding level and genetic (deleterious mutation) loads analyses for the southwest China and south China populations

We utilized NeEstimator v.2.1 (Do et al. 2014) to estimate the effective population sizes (*N*e) of each population during three time periods (pre-1979, 1980-1999, and post-2000). To mitigate the influence of sample size on population genomic data, we first selected an equal number of samples randomly from each time period and then employed an LD-based approach to generate 100 effective population size (Ne) estimates per period.

We assessed the genetic diversity, inbreeding level and deleterious mutation loads of each individual in each population, and compared them within different time periods (pre-1979, 1980–1999, and post-2000). Genetic diversity was assessed by whole genome nucleotide diversity (*π*) using VCFtools with a 50-kb non-overlapping window. We utilized the inbreeding coefficient (*F*_ROH_) to evaluate the inbreeding level. We identified runs of homozygosity (ROHs) exceeding 100 kb using PLINK v.2.0 (Purcell et al. 2007). The parameters for homozyg-window-snp and homozyg-window-het were set to 20 and 1, respectively. ROHs between 100 kb and 1Mb, as well as those longer than 1Mb, were detected in each individual (Hu et al. 2020; Wei et al. 2024; Zhai et al. 2025; Li et al. 2026). *F*_ROH > 100kb_ is calculated by dividing the total length of the ROHs longer than 100 kb by the overall length of the genome compared to the reference genome (McQuillan et al. 2008). We calculated the proportion of homozygous loss of function (LOF) mutations, and deleterious missense mutations of all individuals in each population, to assess the genetic load. Given that the growing evidence from recent studies indicates that synonymous mutations can frequently have functional impacts and are largely non-neutral (Shen et al. 2022; Kruglyak et al. 2023; Niu et al. 2025), we also use synonymous mutations to estimate genetic load. First, the genotypes of major homozygous alleles in Chinese pangolin (>50% of the individuals) and also the same homozygous alleles in the other pangolin species (as the out-group) were used to represent the ancestral state (Feng et al. 2019). Second, we utilized SnpEff v.4.3t (Cingolani et al. 2012) to construct a database based on the reference genome and annotation information for the Chinese pangolin, and annotated individual allelic mutations into different mutation types accordingly. Finally, we defined missense mutations with a Grantham Score (GS) (Grantham 1974) greater than 150 as deleterious missense mutations (Li et al. 1984). We estimated the genetic load of each individual by calculating the ratio of homozygous deleterious mutations to the total number of homozygous and heterozygous deleterious mutations (Robinson et al. 2016).

Due to the number of samples are totally different, the genetic diversity, inbreeding level and deleterious mutation loads of each time period in each population was resampled to re-evaluate and used the median as the final value. We resampled 5 and 10 individuals from each period of south China population and southwest China population, respectively. Each dataset resampled 100 times. All the results were subjected to significance testing using the Wilcoxon rank-sum test method. Proportional changes were calculated as (value_after − value_before)/value_before. The summarized values represent the average across 100 resampling iterations at each time point, and the box plot depicts the quantile-based range of these results.

### Future evolution potential simulation

To assess the future evolutionary potential of south China population and southwest China population, we performed forward genetic simulations over 1000 years for each population separately using SLiM v.3.6 (Haller and Messer 2019). These simulations employed a non-Wright-Fisher (nonWF) model, parameterized by the historical diversity levels and distinct demographic histories which simulated the effective population sizes (*Ne*) of each population across three time periods (pre-1979, 1980–1999, and post-2000) using NeEstimator v.2.1 (Do et al. 2014). Utilizing the genomic characteristics of the Chinese pangolin, we constructed a genome comprising 19 chromosomes and 20,000 genes, each with a length of 1500 bp (Xie et al. 2022). Genes were assigned to chromosomes in proportion to the length of each chromosome (Xie et al. 2022). The generation time of the Chinese pangolin was set at one year (Zhang et al. 2016), and the mutation rate was set at 1.47 × 10^–8^ per site per generation (Choo et al. 2016). The recombination rate between genes was set to 1 × 10^–3^ per site per generation, and the ratio of non-synonymous mutations to synonymous mutations was set to 2.31:1 (Huber et al. 2017). The selection coefficient (*s*) and dominance coefficient (*h*) for deleterious mutations were determined according to the distribution of human fitness effects (Kim et al. 2017), when *s*<–0.01, *h* = 0, and when *s*≥–0.01, *h* = 0.25 (Kyriazis et al. 2020). Each generation was assigned a random mortality rate based on to stochastic events, and the age at which individuals first reproduce was set at one year. We ran each simulation with an ancestral carrying capacity of 10,000 for 100,000 generations for the burn-in process.

The carrying capacity (*K*) value was fixed from the start of each scenario because numerous external factors can influence its trajectory, and simulating variable *K* value would make results difficult to interpret. To evaluate model sensitivity to alternative trajectories of the *K*, we specified a series of step-change scenarios. A declining series (50, 40, 30, 20, 10) modeled a pessimistic scenario where current conservation efforts are insufficient, leading to continued population decline, thereby allowing assessment of extinction risk under unfavorable conditions. Conversely, an increasing series (100, 150, 200, 250) reflected an optimistic scenario in which proactive, effective measures (such as those currently implemented by the Chinese government in key habitats) successfully enhance habitat quality and promote population recovery. These ranges enabled evaluation across different conservation futures. We defined extinction probability as the proportion of population extinctions after 100 simulations and extinction time as the point when population size reached zero. Deleterious mutations were classified as strong (s ≤ –0.01), moderate (−0.01 < s ≤ –0.001), or weak (−0.001 < s ≤ –0.00001) following Dussex et al. (2021). Population mean fitness was calculated as the proportion of homozygous deleterious mutations among all individuals.

## Supplementary Material

msag099_Supplementary_Data

## Data Availability

The resequencing short-read Fastq files generated in this study have been deposited in the CNGBdb (https://db.cngb.org/) archive under project CNP0007284. The analyses codes have been made publicly available in figshare (10.6084/m9.figshare.30737891).
