# Supplementary material for: Pangolin museum genomics reveal the dynamic genetic consequences and extinction risk of the critically endangered Chinese pangolin
Source: Mol Biol Evol. 2026 Apr 9;43(5):msag099. doi: 10.1093/molbev/msag099 (PMC13137990; doi:10.1093/molbev/msag099)
Supplement: msag099_Supplementary_Data [file msag099_supplementary_data.pdf]

Pangolin museum genomics reveal the dynamic genetic consequences and extinction risk of the critically endangered Chinese pangolin

Jing Yang Hu[1,*], Yu Jiang[1], Song Li[2], Ting Ting Ying[3], Li Yu[1,4,*]

[1] School of Life Sciences and State Key Laboratory for Conservation and Utilization of Bio-Resources in Yunnan, Yunnan University, Kunming 650091, China

[2] Kunming Natural History Museum of Zoology, Kunming Institute of Zoology, Chinese Academy of Sciences, Kunming 650221, China

[3] State Key Laboratory of Genetic Resources and Evolution, Kunming Institute of Zoology, Chinese Academy of Sciences, Kunming 650233, China

[4] Southwest United Graduate School, Kunming 650091, China

[*]**Corresponding author:** Li Yu, Jing Yang Hu

E-mail: yuli@ynu.edu.cn; hujingyang@ynu.edu.cn

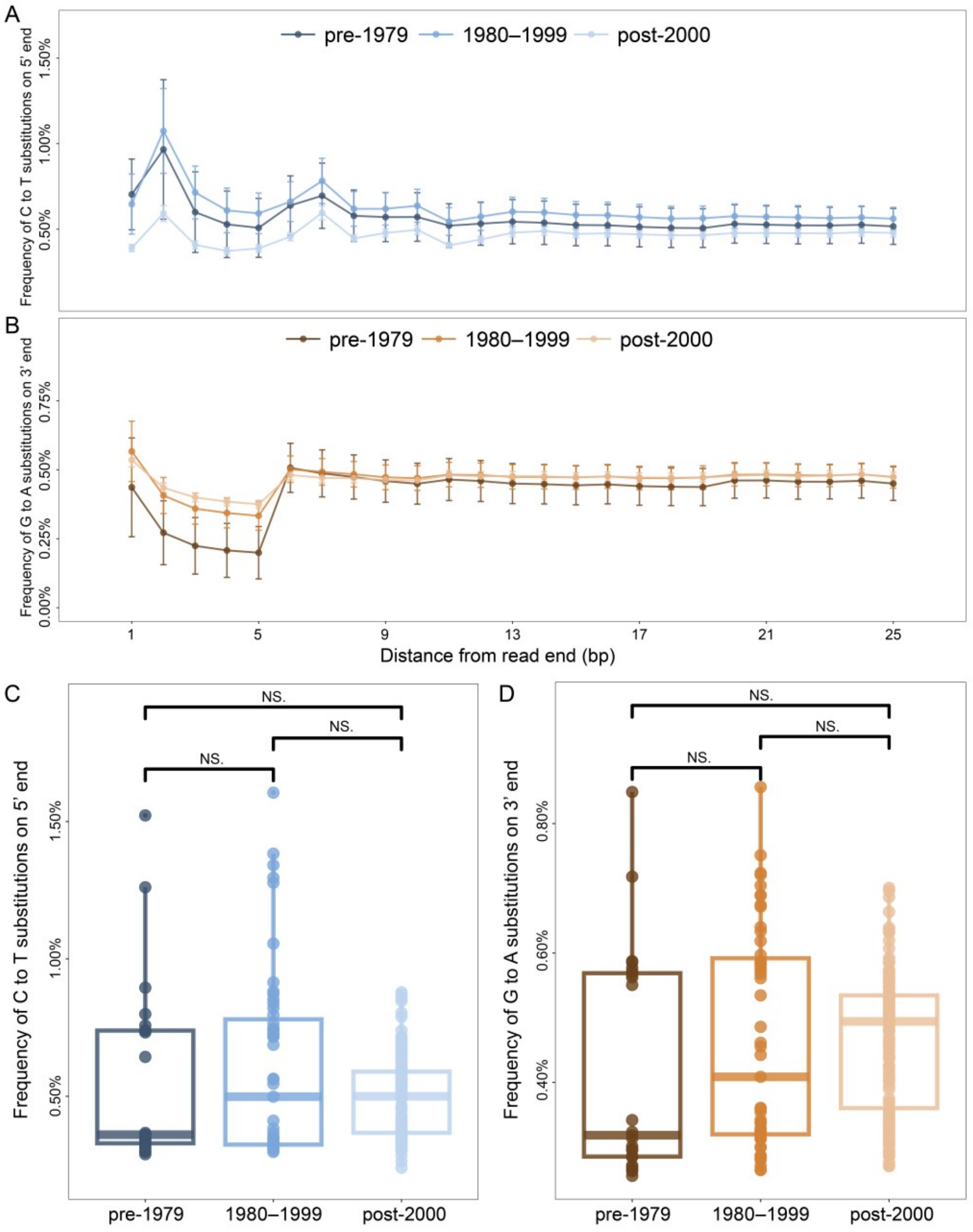

Supplementary Figure S1. DNA damage and temporal genomics of the Chinese pangolin.

(A) The average frequency of C (Cytosine) to T (Thymine) substitutions at the 5' end, by distance. The vertical bars represent the 95% confidence intervals. (B) The average frequency of G (Guanine) to A (Adenine) substitutions at the 3' end, by distance. The vertical bars represent the 95% confidence intervals. (C) The average frequency of C to T substitutions at the 5' end, during different periods. "NS." indicates the *P* value was not significant. (D) The average frequency of G to A substitutions at the 3' end, during different periods. "NS." indicates the *P* value was not significant.

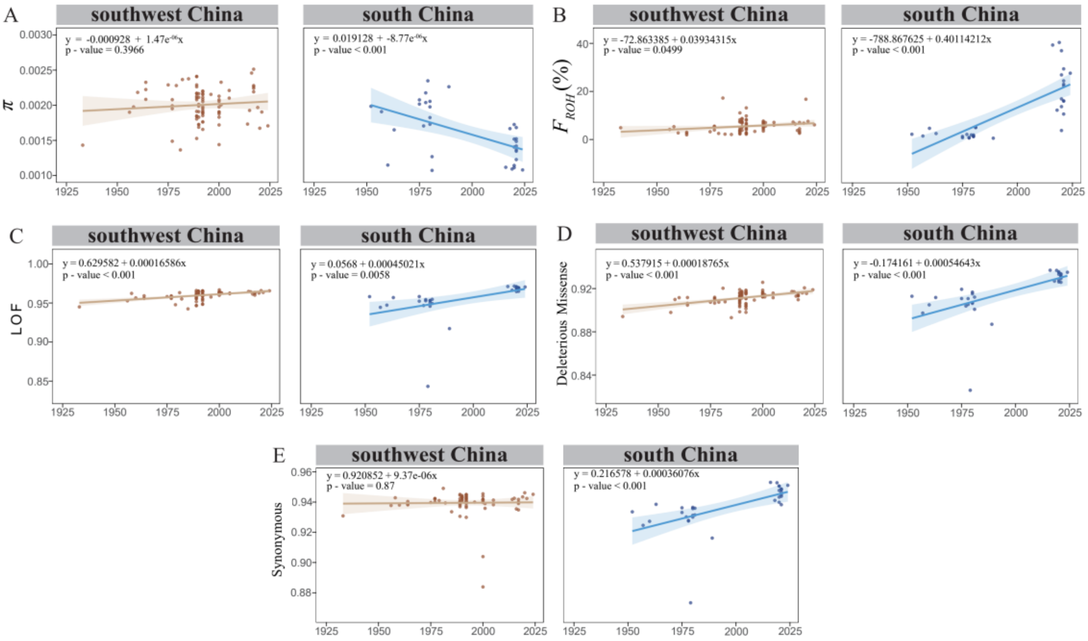

Supplementary Figure S2. Dynamic genetic consequences of southwest China and south China populations of Chinese pangolin correlating with the exact sampling time of each sample.

(A)nucleotide diversity (π), (B) inbreeding coefficient ($F_{ROH>100kb}$), (C) genetic load of loss of function (LOF) mutations, (D) genetic load of deleterious missense mutations, and (E) genetic load of synonymous mutations. Each result was fitted with a linear regression trend line, slope significance was tested with two-tailed t-test and significance was set at p = 0.05 (95% CI for slope was also reported).

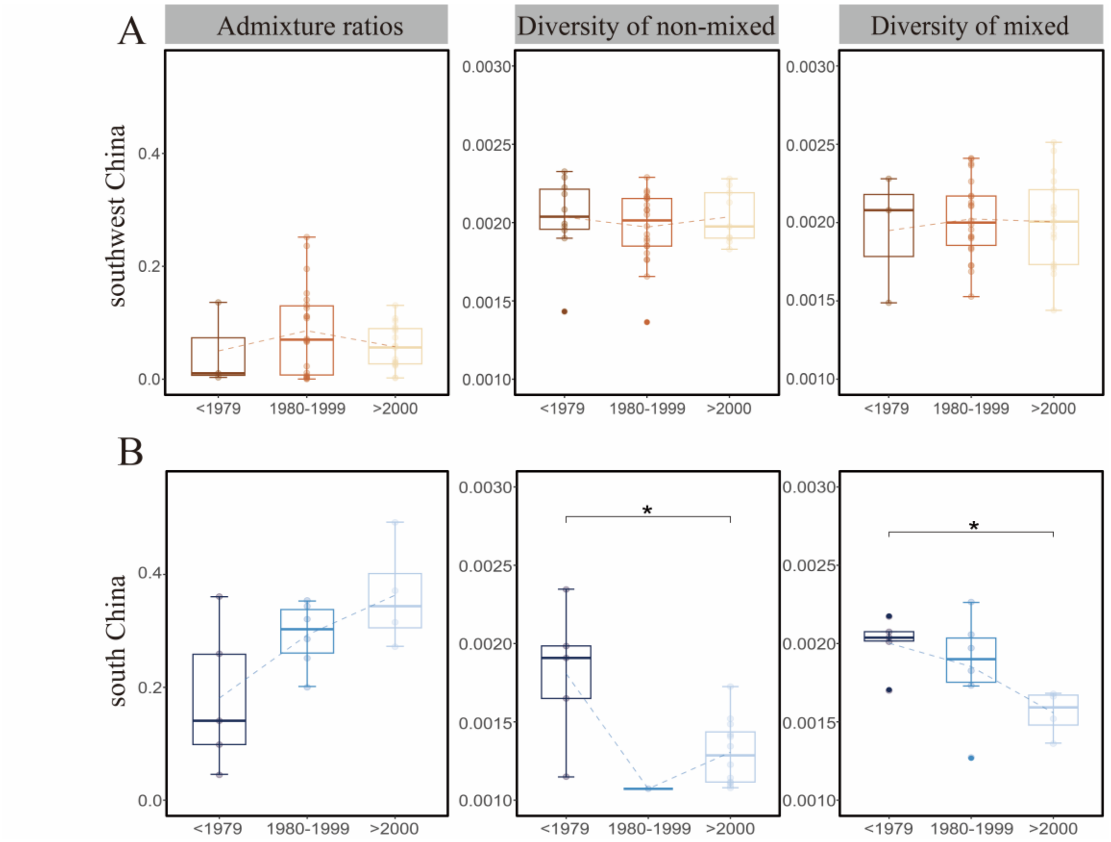

Supplementary Figure S3. Comparison of the admixture ratios and genetic diversity of southwest China (A) and south China (B) populations of Chinese pangolin correlating with different sampling time. Admixture ratios represent the strength of gene flow or mixed ratios determined by Dystruct. Diversity of non-mixed represented the average diversity of non-mixed individual. Diversity of mixed represented the average diversity of mixed individual. The results of the significance test are presented in the figure. The * represent P values less than 0.05.

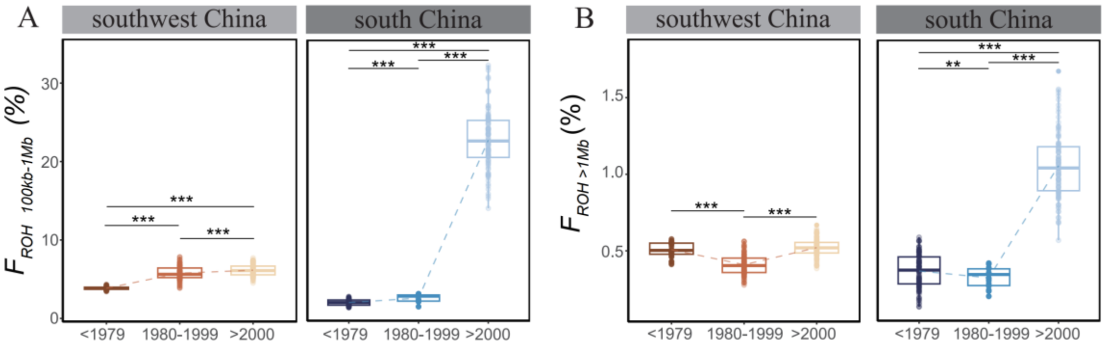

Supplementary Figure S4. Showing ROH length distribution. The ROH length distribution is calculated by dividing the total length of the ROHs between 100kb and 1Mb (A), as well as those longer than 1Mb (B), by the overall length of the genome compared to the reference genome, respectively.

Supplementary Table S1. Sample information and sequencing data.

Sample ID	Previous ID	Location	sample types	Sampling time (year)	Clean data (Gb)	Mapping depth (×)	Mapping rate (%)	Genetic population	Resources
MP01	MP01	Yunnan, China	/	1990	119.10	31.57	99.46	Southwest China	Hu et al. 2020
MP02	MP02	Yunnan, China	/	1992	105.27	31.40	99.42	Southwest China	Hu et al. 2020
MP03	MP03	Yunnan, China	/	2000	110.65	30.06	99.63	Southwest China	Hu et al. 2020
MP04	MP04	Yunnan, China	/	1992	103.23	31.58	99.48	Southwest China	Hu et al. 2020
MP05	MP05	Yunnan, China	/	2000	130.60	32.30	99.37	Southwest China	Hu et al. 2020
MP06	MP06	Yunnan, China	/	1990	111.00	31.10	99.47	Southwest China	Hu et al. 2020
MP08	MP08	Yunnan, China	/	2000	121.87	31.99	99.49	Southwest China	Hu et al. 2020
MP09	MP09	Yunnan, China	/	2017	115.16	31.29	99.36	Southwest China	Hu et al. 2020
MP10	MP10	Yunnan, China	/	2017	109.07	27.97	99.15	Southwest China	Hu et al. 2020
MP11	MP11	Yunnan, China	/	2005	117.85	30.54	99.68	Southwest China	Hu et al. 2020
MP12	MP12	Yunnan, China	/	2017	96.07	29.22	99.22	Southwest China	Hu et al. 2020
MP13	MP13	Yunnan, China	/	2016	114.89	31.82	99.31	Southwest China	Hu et al. 2020
MP14	MP14	Yunnan, China	/	2017	93.09	28.78	99.37	Southwest China	Hu et al. 2020
MP15	MP15	Unknown	/	2016	116.58	28.51	98.89	Nepal	Hu et al. 2020
MP16	MP16	Unknown	/	2014	101.07	20.73	98.49	Nepal	Hu et al. 2020
MP17	MP17	Unknown	/	2014	96.96	23.02	98.30	Nepal	Hu et al. 2020

MP18	MP18	Unknown	/	2014	117.10	34.70	98.86	Nepal	Hu et al. 2020
MP19	MP19	Unknown	/	2017	104.13	28.68	98.44	Nepal	Hu et al. 2020
MP20	MP20	Unknown	/	2017	143.67	23.14	99.00	Nepal	Hu et al. 2020
MP21	MP21	Unknown	/	2016	103.16	25.91	98.38	Nepal	Hu et al. 2020
MP22	MP22	Unknown	/	2016	128.35	34.41	98.59	Nepal	Hu et al. 2020
MP23	MP23	Unknown	/	2017	91.61	26.56	98.40	Nepal	Hu et al. 2020
MP24	GAFM0001	Unknown	/	2016	32.29	10.60	99.25	Indo-China	Wang et al. 2022
MP25	GAFM0005	Guangdong, China	/	2020	54.83	17.63	99.22	South China	Wang et al. 2022
MP26	GAFM0006	Guangdong, China	/	2020	37.35	12.15	98.93	South China	Wang et al. 2022
MP27	GAFM0008	Unknown	/	2020	41.30	13.41	99.41	Indo-China	Wang et al. 2022
MP28	GAFM0009	Unknown	/	2020	38.31	12.90	99.38	Indo-China	Wang et al. 2022
MP29	GAFM0010	Unknown	/	2020	39.70	13.34	99.44	Indo-China	Wang et al. 2022
MP30	GAFM0012	Unknown	/	2020	40.72	13.74	99.33	Indo-China	Wang et al. 2022

MP31	GAFM0013	Unknown	/	2020	38.32	12.67	99.06	Southwest China	Wang et al. 2022
MP32	GAFM0014	Unknown	/	2019	44.67	14.44	99.40	Indo-China	Wang et al. 2022
MP33	GAFM0015	Unknown	/	2019	44.39	14.53	99.44	Indo-China	Wang et al. 2022
MP34	GAFM0016	Unknown	/	2019	35.72	11.83	99.21	Indo-China	Wang et al. 2022
MP35	GAFM0017	Guangdong, China	/	2019	31.60	10.51	98.81	South China	Wang et al. 2022
MP36	GAFM0019	Unknown	/	2019	41.31	13.43	99.43	Indo-China	Wang et al. 2022
MP37	GAFM0020	Unknown	/	2020	49.12	14.94	99.42	Indo-China	Wang et al. 2022
MP38	GAFM0021	Unknown	/	2020	43.41	14.36	99.38	Indo-China	Wang et al. 2022
MP39	MP39	Unknown	/	2020	49.24	16.11	99.31	Indo-China	Wei et al. 2024
MP40	MP40	Guangdong, China	/	2016	59.05	20.13	99.10	South China	Wei et al. 2024
MP41	MP41	Unknown	/	2020	57.36	18.43	99.56	Indo-China	Wei et al. 2024
MP42	MP42	Guangdong, China	/	2020	82.86	27.03	99.30	South China	Wei et al. 2024

MP43	MP43	Unknown	/	2020	75.37	25.05	99.52	Indo-China	Wei et al. 2024
MP44	MP44	Unknown	/	2020	73.30	23.80	99.53	Indo-China	Wei et al. 2024
MP45	MP45	Unknown	/	2019	46.42	7.68	98.03	Indo-China	Wei et al. 2024
MP46	MP46	Vietnam	/	2021	33.16	11.64	98.91	Indo-China	Wei et al. 2024
MP47	MP47	Unknown	/	2021	33.19	11.56	98.66	Southwest China	Wei et al. 2024
MP48	MP48	Unknown	/	2021	32.31	11.66	98.82	Indo-China	Wei et al. 2024
MP49	MP49	Unknown	/	2021	32.72	11.25	98.83	Indo-China	Wei et al. 2024
MP50	MP50	Unknown	/	2021	28.61	10.03	99.15	Indo-China	Wei et al. 2024
MP51	MP51	Unknown	/	2021	30.68	10.63	98.98	Indo-China	Wei et al. 2024
MP52	MP52	Jiangxi, China	/	2021	33.72	11.85	98.42	South China	Wei et al. 2024
MP53	MP53	Guangdong, China	/	2021	31.67	11.20	98.36	South China	Wei et al. 2024
MP54	MP54	Guangdong, China	/	2021	31.93	10.98	98.50	South China	Wei et al. 2024
MP55	MP55	Guangdong, China	/	2021	33.26	11.75	98.35	South China	Wei et al. 2024
MP56	MP56	Guangdong, China	/	2021	30.90	10.65	98.43	South China	Wei et al. 2024
MP57	MP57	Fujian, China	/	2021	36.91	13.03	98.37	South China	Wei et al. 2024
MP58	MP58	Anhui, China	/	2021	31.98	11.36	98.44	South China	Wei et al. 2024
MP59	MP59	Vietnam	/	2021	93.56	31.40	99.84	Indo-China	Wei et al. 2024
MP60	MP60	Yunnan, China	/	1992	151.57	16.36	99.37	Southwest China	Wei et al. 2024
MP61	MP61	Yunnan, China	/	1989	80.45	17.46	99.39	Southwest China	Wei et al. 2024
MP62	MP62	Yunnan, China	/	1985	80.93	17.71	99.29	Southwest China	Wei et al. 2024

MP63	MP63	Yunnan, China	/	1964	80.85	17.91	99.23	Southwest China	Wei et al. 2024
MP64	MP64	Yunnan, China	/	1992	81.27	13.59	99.21	Southwest China	Wei et al. 2024
MP65	MP65	Yunnan, China	/	1964	80.24	11.67	95.31	Southwest China	Wei et al. 2024
MP66	MP66	Sichuan, China	/	1975	104.86	15.01	99.53	Southwest China	Wei et al. 2024
MP67	MP67	Yunnan, China	/	1989	87.91	10.67	99.18	Southwest China	Wei et al. 2024
MP68	MP68	Yunnan, China	/	1989	122.14	10.25	98.68	Southwest China	Wei et al. 2024
MP69	MP69	Hainan, China	/	1960	109.82	7.84	93.64	South China	Wei et al. 2024
MP70	MP70	Yunnan, China	/	1992	98.65	10.38	97.34	Southwest China	Wei et al. 2024
MP71	MP71	Yunnan, China	/	1981	85.47	17.80	99.08	Southwest China	Wei et al. 2024
MP72	MP72	Hainan, China	/	1957	94.73	10.84	98.21	South China	Wei et al. 2024
MP73	MP73	Yunnan, China	/	1958	80.01	15.84	99.46	Southwest China	Wei et al. 2024
MP74	MP74	Hainan, China	/	1963	115.32	17.74	99.28	South China	Wei et al. 2024
MP75	MP75	Yunnan, China	/	1933	107.27	6.42	93.05	Southwest China	Wei et al. 2024
MP76	MP76	Yunnan, China	/	1956	99.39	11.61	98.65	Southwest China	Wei et al. 2024
MP77	MP77	Hunan, China	/	1979	114.30	9.91	97.48	South China	Wei et al. 2024
MP78	MP78	Sichuan, China	/	1977	86.31	10.10	97.91	South China	Wei et al. 2024
MP79	MP79	Zhejiang, China	/	1981	80.78	7.40	89.51	South China	Wei et al. 2024
MP80	MP80	Anhui, China	/	2020	106.52	17.99	99.41	South China	Wei et al. 2024
MP81	MP81	Guangxi, China	/	1952	128.77	20.41	99.40	South China	Wei et al. 2024
MP82	MP82	Guizhou, China	/	1980	87.21	11.48	98.98	South China	Wei et al. 2024

MP83	MP83	Guizhou, China	/	1978	114.05	13.34	99.16	South China	Wei et al. 2024
MP84	MP84	Guizhou, China	/	1975	111.42	12.75	99.19	South China	Wei et al. 2024
MP85	MP85	Guizhou, China	/	1978	101.51	13.33	98.22	South China	Wei et al. 2024
MP86	MP86	Guizhou, China	/	1980	80.54	10.54	98.47	South China	Wei et al. 2024
MP87	MP87	Guizhou, China	/	1980	122.13	10.19	98.30	South China	Wei et al. 2024
MP88	MP88	Guizhou, China	/	1975	89.07	13.70	99.06	South China	Wei et al. 2024
MP89	MP89	Guizhou, China	/	1977	80.93	8.37	93.36	Southwest China	Wei et al. 2024
MP90	MP90	Guizhou, China	/	1980	83.54	9.70	97.47	South China	Wei et al. 2024
MP91	MP91	Guizhou, China	/	1981	97.40	9.65	89.47	South China	Wei et al. 2024
MP92	MP92	Vietnam-China border	/	2020	97.71	22.86	99.58	Indo-China	Wei et al. 2024
MP93	MP93	Guangxi, China	/	2020	82.59	21.17	99.32	South China	Wei et al. 2024
MP94	MP94	Yunnan, China	/	2019	86.84	20.91	99.38	Southwest China	Wei et al. 2024
MP95	MP95	Zhejiang, China	/	2024	32.28	8.97	98.54	South China	Zhai et al. 2025
MP96	MP96	Hainan, China	/	2018	90.44	25.47	99.21	South China	Zhai et al. 2025
MP97	/	Unknown	scale	2015	31.33	8.80	97.53	Sino-Burmese	This study
MP98	/	Unknown	scale	2015	33.80	8.69	97.54	Nepal	This study
MP99	/	Unknown	scale	2015	40.01	9.97	97.80	Sino-Burmese	This study
MP100	/	Unknown	scale	2015	31.92	6.16	96.74	Sino-Burmese	This study
MP101	/	Unknown	scale	2015	32.20	7.47	98.73	Southwest China	This study

MP102	/	Unknown	scale	2014	41.24	12.63	97.43	Nepal	This study
MP103	/	Unknown	scale	2019	42.38	12.24	97.77	Nepal	This study
MP104	/	Unknown	scale	2019	36.71	10.34	97.60	Nepal	This study
MP105	/	Unknown	scale	2019	34.99	10.36	97.52	Nepal	This study
MP106	/	Unknown	scale	2019	45.01	13.08	97.57	Nepal	This study
MP107	/	Unknown	scale	2019	45.12	11.71	97.75	Nepal	This study
MP108	/	Unknown	scale	2019	32.48	8.63	97.58	Nepal	This study
MP109	/	Unknown	scale	2019	34.98	7.54	97.15	Nepal	This study
MP110	/	Unknown	scale	2019	40.10	11.23	97.71	Nepal	This study
MP111	/	Unknown	scale	2016	48.84	13.29	97.81	Nepal	This study
MP112	/	Unknown	scale	2016	32.40	8.60	97.28	Nepal	This study
MP113	/	Unknown	scale	2016	36.67	10.06	97.78	Nepal	This study
MP114	/	Unknown	scale	2016	41.54	12.00	97.82	Nepal	This study
MP115	/	Unknown	scale	2018	31.30	8.09	97.58	Nepal	This study
MP116	/	Unknown	scale	2012	40.03	10.79	97.81	Sino-Burmese	This study
MP117	/	Unknown	scale	2012	36.91	10.10	97.73	Sino-Burmese	This study
MP118	/	Unknown	scale	2012	39.32	10.27	97.72	Sino-Burmese	This study
MP119	/	Unknown	scale	2012	42.55	10.95	97.95	Sino-Burmese	This study
MP120	/	Unknown	scale	2012	37.44	10.14	97.93	Sino-Burmese	This study
MP121	/	Unknown	scale	2014	32.81	9.38	97.51	Nepal	This study

MP122	/	Unknown	scale	2014	35.88	9.56	97.71	Nepal	This study
MP123	/	Unknown	scale	2014	40.64	11.08	97.85	Nepal	This study
MP124	/	Unknown	scale	2014	38.83	10.69	97.91	Nepal	This study
MP125	/	Unknown	scale	2014	37.87	10.46	97.80	Nepal	This study
MP126	/	Unknown	scale	2014	32.46	8.18	97.43	Nepal	This study
MP127	/	Unknown	scale	2018	33.27	8.95	97.50	Sino-Burmese	This study
MP128	/	Unknown	scale	2015	37.75	10.45	97.80	Nepal	This study
MP129	/	Unknown	scale	2015	33.05	9.17	97.63	Nepal	This study
MP130	/	Unknown	scale	2015	34.92	9.82	97.84	Nepal	This study
MP131	/	Yunnan, China	skin	1960	34.58	9.53	98.77	Southwest China	This study
MP132	/	Yunnan, China	skin	1964	95.66	16.33	99.31	Southwest China	This study
MP133	/	Yunnan, China	skin	1977	48.26	11.00	99.14	Southwest China	This study
MP134	/	Yunnan, China	skin	1977	61.70	14.25	99.14	Southwest China	This study
MP135	/	Yunnan, China	skin	1977	41.42	9.62	98.94	Southwest China	This study
MP136	/	Yunnan, China	skin	1979	42.70	9.01	98.53	Southwest China	This study
MP137	/	Yunnan, China	skin	1982	76.20	15.13	99.52	Southwest China	This study
MP138	/	Yunnan, China	skin	1989	88.44	17.90	99.41	Southwest China	This study
MP139	/	Unknown	skin	1989	75.61	8.71	98.62	South China	This study
MP140	/	Yunnan, China	skin	1989	85.06	19.42	99.29	Southwest China	This study
MP141	/	Yunnan, China	skin	1989	50.78	11.51	99.08	Southwest China	This study

MP142	/	Yunnan, China	skin	1989	59.31	14.07	99.34	Southwest China	This study
MP143	/	Yunnan, China	skin	1989	97.62	19.70	99.43	Southwest China	This study
MP144	/	Yunnan, China	skin	1989	68.15	15.20	99.39	Southwest China	This study
MP145	/	Yunnan, China	skin	1989	63.05	15.09	99.20	Southwest China	This study
MP146	/	Yunnan, China	skin	1989	78.86	18.61	99.30	Southwest China	This study
MP147	/	Yunnan, China	skin	1989	86.80	20.86	99.43	Southwest China	This study
MP148	/	Yunnan, China	skin	1990	52.34	12.66	99.36	Southwest China	This study
MP149	/	Yunnan, China	skin	1990	70.13	15.33	99.31	Southwest China	This study
MP150	/	Yunnan, China	skin	1992	65.09	13.47	99.18	Southwest China	This study
MP151	/	Yunnan, China	skin	1992	47.89	9.17	99.06	Southwest China	This study
MP152	/	Yunnan, China	skin	1992	57.22	14.15	99.35	Southwest China	This study
MP153	/	Yunnan, China	skin	1992	44.52	11.09	99.05	Southwest China	This study
MP154	/	Yunnan, China	skin	1992	53.39	12.22	99.23	Southwest China	This study
MP155	/	Yunnan, China	skin	1992	48.26	11.35	98.85	Southwest China	This study
MP156	/	Yunnan, China	skin	1992	57.40	12.90	99.26	Southwest China	This study
MP157	/	Yunnan, China	skin	1996	52.77	12.97	99.29	Southwest China	This study
MP158	/	Yunnan, China	skin	2005	45.28	11.90	99.04	Southwest China	This study
MP159	/	Unknown	skin	2019	45.34	12.13	99.16	Indo-China	This study
MP160	/	Unknown		2019	32.81	9.86	99.20	Indo-China	This study
MP161	/	Unknown	scale	2016	42.91	9.93	98.18	Nepal	This study

MP162	/	Unknown	scale	2016	31.76	8.19	97.76	Nepal	This study
MP163	/	Unknown	scale	2017	54.06	12.61	98.09	Sino-Burmese	This study
MP164	/	Unknown	scale	2017	30.24	7.57	97.08	Sino-Burmese	This study
MP165	/	Unknown	scale	2017	51.39	15.15	97.99	Sino-Burmese	This study
MP166	/	Unknown	scale	2017	43.39	12.39	97.96	Sino-Burmese	This study
MP167	/	Unknown	scale	2017	40.81	10.96	97.92	Sino-Burmese	This study
MP168	/	Unknown	scale	2017	51.38	11.17	97.77	Sino-Burmese	This study
MP169	/	Unknown	scale	2017	30.15	8.01	97.23	Sino-Burmese	This study
MP170	/	Unknown	scale	2017	52.92	14.73	98.11	Sino-Burmese	This study
MP171	/	Unknown	scale	2017	32.20	9.28	97.67	Nepal	This study
MP172	/	Unknown	scale	2017	36.48	9.77	97.90	Nepal	This study
MP173	/	Unknown	scale	2017	49.57	13.93	99.09	Southwest China	This study
MP174	/	Unknown	scale	2017	39.80	12.59	97.83	Nepal	This study
MP175	/	Unknown	scale	2017	34.85	10.61	97.75	Sino-Burmese	This study
MP176	/	Unknown	scale	2017	52.62	15.25	98.12	Sino-Burmese	This study
MP177	/	Unknown	scale	2017	33.87	9.31	97.62	Sino-Burmese	This study
MP178	/	Unknown	scale	2017	33.75	8.95	97.47	Sino-Burmese	This study
MP179	/	Unknown	scale	2016	75.31	18.42	98.08	Nepal	This study
MP180	/	Unknown	scale	2016	73.17	18.44	98.30	Nepal	This study
MP181	/	Unknown	scale	2016	115.70	32.72	98.59	Nepal	This study

MP182	/	Unknown	scale	2016	66.51	17.67	98.29	Nepal	This study
MP183	/	Unknown	scale	2016	48.24	12.98	97.93	Nepal	This study
MP184	/	Unknown	scale	2016	33.46	9.60	97.58	Nepal	This study
MP185	/	Unknown	scale	2016	63.46	12.85	97.96	Nepal	This study
MP186	/	Unknown	scale	2016	62.21	18.23	98.19	Nepal	This study
MP187	/	Unknown	scale	2016	38.82	10.49	97.77	Nepal	This study
MP188	/	Unknown	scale	2016	37.51	8.15	97.48	Nepal	This study
MP189	/	Unknown	scale	2015	31.44	7.42	91.45	Sino-Burmese	This study
MP190	/	Unknown	scale	2015	92.88	25.92	98.43	Sino-Burmese	This study
MP191	/	Unknown	scale	2017	31.69	8.71	96.95	Sino-Burmese	This study
MP192	/	Unknown	scale	2017	66.19	12.62	97.22	Sino-Burmese	This study
MP193	/	Unknown	scale	2017	48.88	13.26	97.85	Sino-Burmese	This study
MP194	/	Unknown	scale	2017	35.81	9.20	96.94	Sino-Burmese	This study
MP195	/	Unknown	scale	2017	29.52	8.31	97.46	Sino-Burmese	This study
MP196	/	Unknown	scale	2017	56.06	14.14	98.15	Sino-Burmese	This study
MP197	/	Unknown	scale	2017	56.14	9.44	97.69	Sino-Burmese	This study
MP198	/	Unknown	scale	2017	51.95	11.44	98.24	Sino-Burmese	This study
MP199	/	Unknown	scale	2017	30.96	8.74	97.59	Sino-Burmese	This study
MP200	/	Unknown	scale	2017	33.19	8.84	97.53	Sino-Burmese	This study
MP201	/	Unknown	scale	2024	26.22	7.12	98.66	Southwest China	This study

MP202	/	Unknown	scale	2024	33.02	8.67	98.91	Indo-China	This study
MP203	/	Unknown	scale	2019	75.92	21.57	99.17	Indo-China	This study
MP204	/	Unknown	scale	2016	30.63	8.20	91.01	Sino-Burmese	This study
MP205	/	Yunnan, China	muscle	1992	44.05	10.28	99.27	Southwest China	This study
MP206	/	Yunnan, China	muscle	1992	37.57	9.75	98.93	Southwest China	This study
MP207	/	Yunnan, China	muscle	1992	51.57	11.97	99.22	Southwest China	This study
MP208	/	Yunnan, China	muscle	1992	43.72	10.20	98.90	Southwest China	This study
MP209	/	Yunnan, China	muscle	1992	36.35	9.20	99.01	Southwest China	This study
MP210	/	Yunnan, China	muscle	1989	31.69	7.58	98.77	Southwest China	This study
MP211	/	Yunnan, China	muscle	1989	43.89	10.77	98.86	Southwest China	This study
MP212	/	Yunnan, China	muscle	1990	35.28	9.20	98.92	Southwest China	This study
MP213	/	Yunnan, China	muscle	1990	46.22	11.37	99.33	Southwest China	This study
MP214	/	Yunnan, China	muscle	1990	31.29	8.34	98.89	Southwest China	This study
MP215	/	Yunnan, China	muscle	1990	40.83	10.98	98.98	Southwest China	This study
MP216	/	Yunnan, China	muscle	1990	34.52	8.97	98.97	Southwest China	This study
MP217	/	Unknown	muscle	2000	38.16	8.77	97.96	Nepal	This study
MP218	/	Yunnan, China	muscle	2000	34.89	8.90	98.94	Southwest China	This study
MP219	/	Yunnan, China	muscle	2000	35.15	9.28	98.98	Southwest China	This study
MP220	/	Unknown	muscle	2001	48.67	11.88	99.25	Southwest China	This study
MP221	/	Unknown	muscle	2000	41.72	7.71	98.78	Southwest China	This study

MP222	/	Yunnan, China	muscle	2000	30.57	7.69	98.44	Southwest China	This study
MP223	/	Yunnan, China	muscle	2000	32.55	8.02	98.82	Southwest China	This study
MP224	/	Yunnan, China	muscle	2000	32.96	8.87	98.97	Southwest China	This study
MP225	/	Yunnan, China	muscle	2000	32.97	6.11	97.01	Southwest China	This study
MP226	/	Yunnan, China	muscle	2001	33.09	9.18	98.88	Southwest China	This study
MP227	/	Unknown	muscle	2014	36.27	9.76	98.77	Southwest China	This study
MP228	/	Unknown	muscle	2019	32.21	6.04	95.36	Nepal	This study
MP229	/	Unknown	muscle	2019	39.71	10.02	97.89	Nepal	This study
Outgroup	PTR02	Unknown	/	Unknown	100.89	21.37	92.18	White-bellied pangolin	Gu et al. 2023

Supplementary Table S2. Genetic diversity, inbreeding level and realized load of Chinese pangolins.

Sample	Genetic population	Sampling time (year)	π	F_{ROH}* (%)	Realized load (ratio of 2*homo/(2*homo+hete))		
					Loss of function mutations	Deleterious missense mutations	Synonymous mutations
MP01	Southwest China	1990	0.0021	5.37	0.96	0.91	0.94
MP02	Southwest China	1992	0.0021	6.13	0.96	0.92	0.94
MP03	Southwest China	2000	0.0021	5.59	0.96	0.91	0.88
MP04	Southwest China	1992	0.0020	8.53	0.96	0.92	0.94
MP05	Southwest China	2000	0.0021	5.24	0.96	0.91	0.90
MP06	Southwest China	1990	0.0020	7.06	0.96	0.92	0.94
MP08	Southwest China	2000	0.0021	4.44	0.96	0.91	0.94
MP09	Southwest China	2017	0.0023	3.21	0.96	0.91	0.94
MP10	Southwest China	2017	0.0022	2.77	0.96	0.91	0.94
MP11	Southwest China	2005	0.0021	6.38	0.96	0.92	0.94
MP12	Southwest China	2017	0.0022	7.37	0.96	0.91	0.94
MP13	Southwest China	2016	0.0025	4.03	0.96	0.91	0.94
MP14	Southwest China	2017	0.0025	3.83	0.96	0.91	0.93
MP31	Southwest China	2020	0.0017	17.88	0.97	0.92	0.95

MP47	Southwest China	2021	0.0019	8.32	0.96	0.92	0.94
MP60	Southwest China	1992	0.0019	5.14	0.96	0.90	0.94
MP61	Southwest China	1989	0.0023	2.98	0.96	0.91	0.94
MP62	Southwest China	1985	0.0024	2.34	0.94	0.89	0.93
MP63	Southwest China	1964	0.0022	3.29	0.96	0.90	0.94
MP64	Southwest China	1992	0.0022	2.34	0.95	0.90	0.94
MP65	Southwest China	1964	0.0021	2.25	0.96	0.91	0.94
MP66	Southwest China	1975	0.0023	3.66	0.96	0.91	0.94
MP67	Southwest China	1989	0.0018	4.15	0.95	0.90	0.94
MP68	Southwest China	1989	0.0019	5.18	0.95	0.90	0.93
MP70	Southwest China	1992	0.0018	5.55	0.95	0.90	0.93
MP71	Southwest China	1981	0.0014	17.42	0.96	0.92	0.95
MP73	Southwest China	1958	0.0020	5.81	0.96	0.91	0.94
MP75	Southwest China	1933	0.0014	5.94	0.94	0.89	0.93
MP76	Southwest China	1956	0.0019	5.10	0.95	0.90	0.94
MP89	Southwest China	1977	0.0015	6.26	0.96	0.91	0.94
MP94	Southwest China	2019	0.0020	7.07	0.96	0.92	0.94
MP101	Southwest China	2015	0.0019	7.03	0.96	0.92	0.94
MP131	Southwest China	1960	0.0022	3.14	0.96	0.91	0.94
MP132	Southwest China	1964	0.0023	2.78	0.96	0.91	0.94

MP133	Southwest China	1977	0.0021	4.30	0.96	0.91	0.94
MP134	Southwest China	1977	0.0020	5.86	0.96	0.91	0.94
MP135	Southwest China	1977	0.0020	5.84	0.96	0.91	0.94
MP136	Southwest China	1979	0.0023	2.79	0.95	0.91	0.94
MP137	Southwest China	1982	0.0024	2.35	0.96	0.91	0.94
MP138	Southwest China	1989	0.0024	2.51	0.96	0.91	0.94
MP140	Southwest China	1989	0.0021	7.45	0.96	0.91	0.94
MP141	Southwest China	1989	0.0021	3.67	0.96	0.91	0.94
MP142	Southwest China	1989	0.0023	3.28	0.96	0.91	0.94
MP143	Southwest China	1989	0.0022	3.76	0.96	0.91	0.94
MP144	Southwest China	1989	0.0024	2.56	0.95	0.90	0.94
MP145	Southwest China	1989	0.0018	8.57	0.96	0.92	0.94
MP146	Southwest China	1989	0.0021	5.20	0.96	0.91	0.94
MP147	Southwest China	1989	0.0022	3.99	0.96	0.91	0.94
MP148	Southwest China	1990	0.0020	5.88	0.96	0.91	0.94
MP149	Southwest China	1990	0.0022	4.14	0.96	0.91	0.94
MP150	Southwest China	1992	0.0022	2.79	0.96	0.91	0.94
MP151	Southwest China	1992	0.0022	2.84	0.95	0.90	0.94
MP152	Southwest China	1992	0.0021	4.20	0.96	0.91	0.94
MP153	Southwest China	1992	0.0019	5.95	0.96	0.92	0.94

MP154	Southwest China	1992	0.0022	2.90	0.95	0.91	0.94
MP155	Southwest China	1992	0.0021	2.99	0.96	0.91	0.94
MP156	Southwest China	1992	0.0020	6.46	0.96	0.92	0.94
MP157	Southwest China	1996	0.0022	3.46	0.96	0.91	0.94
MP158	Southwest China	2005	0.0022	7.75	0.96	0.91	0.94
MP173	Southwest China	2017	0.0023	4.98	0.96	0.91	0.94
MP201	Southwest China	2024	0.0017	6.78	0.97	0.92	0.95
MP205	Southwest China	1992	0.0019	7.13	0.96	0.92	0.94
MP206	Southwest China	1992	0.0019	7.31	0.97	0.92	0.95
MP207	Southwest China	1992	0.0019	8.84	0.97	0.91	0.94
MP208	Southwest China	1992	0.0018	10.36	0.97	0.92	0.94
MP209	Southwest China	1992	0.0017	13.82	0.97	0.92	0.94
MP210	Southwest China	1989	0.0015	11.42	0.97	0.92	0.95
MP211	Southwest China	1989	0.0018	8.17	0.96	0.92	0.94
MP212	Southwest China	1990	0.0017	9.36	0.97	0.92	0.94
MP213	Southwest China	1990	0.0019	7.44	0.96	0.91	0.94
MP214	Southwest China	1990	0.0017	8.04	0.96	0.92	0.94
MP215	Southwest China	1990	0.0018	8.06	0.96	0.92	0.94
MP216	Southwest China	1990	0.0017	9.02	0.96	0.92	0.94
MP218	Southwest China	2000	0.0019	8.21	0.96	0.92	0.94

MP219	Southwest China	2000	0.0019	7.06	0.96	0.92	0.94
MP220	Southwest China	2001	0.0023	5.93	0.96	0.91	0.94
MP221	Southwest China	2000	0.0018	8.21	0.96	0.92	0.94
MP222	Southwest China	2000	0.0017	5.39	0.96	0.91	0.94
MP223	Southwest China	2000	0.0019	4.07	0.96	0.91	0.94
MP224	Southwest China	2000	0.0020	7.79	0.96	0.92	0.94
MP225	Southwest China	2000	0.0014	7.43	0.97	0.93	0.95
MP226	Southwest China	2001	0.0020	7.64	0.96	0.91	0.94
MP227	Southwest China	2014	0.0017	8.02	0.96	0.92	0.94
MP25	South China	2020	0.0014	14.43	0.97	0.93	0.95
MP26	South China	2020	0.0011	28.15	0.97	0.93	0.95
MP35	South China	2019	0.0011	42.89	0.97	0.94	0.95
MP40	South China	2016	0.0011	41.68	0.97	0.94	0.95
MP42	South China	2020	0.0011	38.85	0.97	0.94	0.95
MP52	South China	2021	0.0013	25.67	0.97	0.93	0.95
MP53	South China	2021	0.0015	17.31	0.97	0.93	0.94
MP54	South China	2021	0.0014	23.30	0.97	0.93	0.95
MP55	South China	2021	0.0015	16.91	0.97	0.93	0.94
MP56	South China	2021	0.0017	11.36	0.96	0.93	0.94
MP57	South China	2021	0.0014	24.20	0.97	0.93	0.95

MP58	South China	2021	0.0012	30.87	0.97	0.93	0.95
MP69	South China	1960	0.0011	5.90	0.95	0.90	0.93
MP72	South China	1957	0.0019	2.19	0.94	0.90	0.92
MP74	South China	1963	0.0016	2.90	0.96	0.91	0.94
MP77	South China	1979	0.0023	2.58	0.84	0.83	0.87
MP78	South China	1977	0.0017	2.65	0.95	0.90	0.93
MP79	South China	1981	0.0011	5.81	0.95	0.90	0.93
MP80	South China	2020	0.0017	4.01	0.96	0.93	0.94
MP81	South China	1952	0.0020	2.40	0.96	0.91	0.93
MP82	South China	1980	0.0021	1.47	0.95	0.91	0.93
MP83	South China	1978	0.0022	0.82	0.95	0.91	0.93
MP84	South China	1975	0.0021	1.09	0.95	0.91	0.93
MP85	South China	1978	0.0020	1.02	0.95	0.91	0.93
MP86	South China	1980	0.0020	1.65	0.95	0.92	0.94
MP87	South China	1980	0.0017	2.30	0.95	0.91	0.93
MP88	South China	1975	0.0020	1.52	0.96	0.92	0.94
MP90	South China	1980	0.0018	1.97	0.95	0.92	0.94
MP91	South China	1981	0.0013	5.69	0.95	0.91	0.94
MP93	South China	2020	0.0015	17.41	0.97	0.93	0.94
MP95	South China	2024	0.0011	28.51	0.97	0.94	0.95

MP96	South China	2018	0.0017	12.69	0.97	0.93	0.94
MP139	South China	1989	0.0023	0.65	0.92	0.89	0.92

*π: Nucleotide diversity; F_{ROH}: Inbreeding coefficient.

Supplementary Table S3. Confidence intervals describing temporal changes in genetic diversity, inbreeding level and realized load of Chinese pangolins.

Genetic consequences	Time / Population	ranges	pre-1979	1980-1999	post-2000	Period I (%)	Period II (%)	All (%)
Ne	Southwest China	maximum	553.60	246.20	130.10	54.63	27.93	67.30
		minimum	253.90	108.50	92.50			
		medians	327.00	148.70	110.85			
	South China	maximum	150.20	117.00	64.30	8.59	40.81	45.89
		minimum	84.20	85.40	54.30			
		medians	110.00	101.2	59.5			
He	Southwest China	maximum	0.002133	0.002128	0.002204	1.12	1.46	0.32
		minimum	0.001945	0.001809	0.001871			
		medians	0.002006	0.001994	0.002008			
	South China	maximum	0.002112	0.00197	0.001535	9.23	21.30	28.56
		minimum	0.001679	0.001574	0.001131			
		medians	0.001916	0.001712	0.001374			
F_{roh}	Southwest China	maximum	4.34	7.85	7.71	31.92	9.73	53.91
		minimum	3.42	3.87	4.53			
		medians	3.86	5.63	6.10			
	South China	maximum	2.70	3.07	32.36	23.30	741.45	937.53
		minimum	1.28	1.41	14.02			

		medians	1.94	2.75	22.66			
		maximum	0.9557	0.9594	0.9633			
	Southwest China	minimum	0.9544	0.9550	0.9624	0.39	0.41	0.80
		medians	0.9557	0.9595	0.9633			
Lof		maximum	0.9561	0.9521	0.9705			
	South China	minimum	0.9268	0.9431	0.9672	0.59	2.37	2.97
		medians	0.9332	0.9446	0.9687			
		maximum	0.9094	0.9163	0.9173			
	Southwest China	minimum	0.9057	0.9070	0.9134	0.45	0.46	0.91
Deleterious Missense		medians	0.9067	0.9111	0.9152			
		maximum	0.9120	0.9115	0.9351			
	South China	minimum	0.8886	0.9027	0.9286	0.77	2.78	3.57
		medians	0.8948	0.9058	0.9317			
		maximum	0.9399	0.9417	0.9401			
	Southwest China	minimum	0.9392	0.9396	0.9354	0.14	0.33	0.19
		medians	0.9394	0.9408	0.9374			
Synonymous		maximum	0.9308	0.9310	0.9480			
	South China	minimum	0.9399	0.9417	0.9401	0.06	1.70	2.32
		medians	0.9259	0.9310	0.9469			
